# National Tunisian Study of Cardiac Implantable Electronic Devices: Design and Protocol for a Nationwide Multicenter Prospective Observational Study

**DOI:** 10.2196/47525

**Published:** 2024-04-08

**Authors:** Sonia Chabrak, Abdeddayem Haggui, Emna Allouche, Sana Ouali, Afef Ben Halima, Slim Kacem, Salma Krichen, Sonia Marrakchi, Wafa Fehri, Mohamed Sami Mourali, Zeineb Jabbari, Manel Ben Halima, Elyes Neffati, Aymen Heraiech, Mehdi Slim, Salem Kachboura, Habib Gamra, Majed Hassine, Sondes Kraiem, Sofien Kammoun, Leila Bezdah, Gouider Jridi, Hatem Bouraoui, Samir Kammoun, Rania Hammami, Rafik Chettaoui, Youssef Ben Ameur, Fares Azaiez, Rami Tlili, Kais Battikh, Hedi Ben Slima, Rim Chrigui, Samia Fazaa, Islem Sanaa, Yassine Ellouz, Mohamed Mosrati, Sami Milouchi, Soumaya Jarmouni, Wacef Ayadi, Malek Akrout, Rabie Razgallah, Wissal Neffati, Meriem Drissa, Selma Charfeddine, Salem Abdessalem, Leila Abid, Lilia Zakhama

**Affiliations:** 1 Pasteur Clinic General and Cardiovascular Clinic of Tunis Tunis Tunisia; 2 Military Hospital Faculty of Medicine of Tunis University of Tunis Tunis Tunisia; 3 Cardiology Department Faculty of Medicine of Tunis, Charles Nicole Hospital University of Tunis Tunis Tunisia; 4 Cardiology Department Faculty of Medicine of Tunis, La Rabta Hospital University of Tunis Tunis Tunisia; 5 Abderrahmen Mami Hospital Faculty of Medicine of Tunis University of Tunis Tunis Tunisia; 6 Essalem Center Sousse Tunisia; 7 Bassetine Clinic Sfax Tunisia; 8 Cardiology Department Versailles Cardiology Center Paris France; 9 Cardiology Department Faculty of Medicine of Tunis, Military Hospital University of Tunis Tunis Tunisia; 10 Cardiology Department Faculty of Medicine of Tunis, Abderrahmen Mami Hospital University of Tunis Tunis Tunisia; 11 Cardiology Department Faculty of Medicine of Sousse, Sahloul Hospital University of Sousse Sousse Tunisia; 12 Cardiology A Department Fattouma Bourguiba Hospital Monastir Tunisia; 13 Cardiology Department Faculty of Medicine of Tunis, Habib Thameur Hospital University of Tunis Tunis Tunisia; 14 Cardiology Department Faculty of Medicine of Sousse, Farhat Hached Hospital University of Sousse Sousse Tunisia; 15 Cardiology Department Faculty of Medicine of Sfax, Hedi Chaker Hospital University of Sfax Sfax Tunisia; 16 Tunisian Society of Cardiology and Cardiovascular Surgery Tunis Tunisia; 17 Cardiology Department Faculty of Medicine of Tunis, Mongi Slim Hospital University of Tunis Tunis Tunisia; 18 Berges du Lac Clinic Medenine Tunisia; 19 Cardiology Department Faculty of Medicine of Tunis, Menzel Bourguiba Hospital University of Tunis Bizerte Tunisia; 20 General & Cardiovascular Clinic Tunis Tunisia; 21 Carthage International Medical Center Monastir Tunisia; 22 Cardiology Department Habib Bourguiba Hospital University of Sfax Medenine Tunisia; 23 DACIMA Consulting Tunis Tunisia; 24 Cardiology Department Hospital of the Interior Force Security University of Tunis Tunis Tunisia

**Keywords:** Tunisia, study, pacemaker, implantable cardioverter defibrillator, cardiac resynchronization therapy, design, complication

## Abstract

**Background:**

In Tunisia, the number of cardiac implantable electronic devices (CIEDs) is increasing, owing to the increase in patient life expectancy and expanding indications. Despite their life-saving potential and a significant reduction in population morbidity and mortality, their increased numbers have been associated with the development of multiple early and late complications related to vascular access, pockets, leads, or patient characteristics.

**Objective:**

The study aims to identify the rate, type, and predictors of complications occurring within the first year after CIED implantation. It also aims to describe the demographic and epidemiological characteristics of a nationwide sample of patients with CIED in Tunisia. Additionally, the study will evaluate the extent to which Tunisian electrophysiologists follow international guidelines for cardiac pacing and sudden cardiac death prevention.

**Methods:**

The Tunisian National Study of Cardiac Implantable Electronic Devices (NATURE-CIED) is a national, multicenter, prospectively monitored study that includes consecutive patients who underwent primary CIED implantation, generator replacement, and upgrade procedure. Patients were enrolled between January 18, 2021, and February 18, 2022, at all Tunisian public and private CIED implantation centers that agreed to participate in the study. All enrolled patients entered a 1-year follow-up period, with 4 consecutive visits at 1, 3, 6, and 12 months after CIED implantation. The collected data are recorded electronically on the clinical suite platform (DACIMA Clinical Suite).

**Results:**

The study started on January 18, 2021, and concluded on February 18, 2023. In total, 27 cardiologists actively participated in data collection. Over this period, 1500 patients were enrolled in the study consecutively. The mean age of the patients was 70.1 (SD 15.2) years, with a sex ratio of 1:15. Nine hundred (60%) patients were from the public sector, while 600 (40%) patients were from the private sector. A total of 1298 (86.3%) patients received a conventional pacemaker and 75 (5%) patients received a biventricular pacemaker (CRT-P). Implantable cardioverter defibrillators were implanted in 127 (8.5%) patients. Of these patients, 45 (3%) underwent CRT-D implantation.

**Conclusions:**

This study will establish the most extensive contemporary longitudinal cohort of patients undergoing CIED implantation in Tunisia, presenting a significant opportunity for real-world clinical epidemiology. It will address a crucial gap in the management of patients during the perioperative phase and follow-up, enabling the identification of individuals at particularly high risk of complications for optimal care.

**Trial Registration:**

ClinicalTrials.gov NCT05361759; https://classic.clinicaltrials.gov/ct2/show/NCT05361759

**International Registered Report Identifier (IRRID):**

RR1-10.2196/47525

## Introduction

### Background

Cardiac implantable electronic devices (CIEDs), including permanent pacemakers (PMs), implantable cardioverter defibrillators (ICDs), and cardiac resynchronization therapy devices with or without defibrillators (CRT-Ds or CRT-Ps), are standard therapy for bradyarrhythmia, tachyarrhythmia, and systolic heart failure [1,2]. Their use has increased over the last decade [3,4]. However, CIED implantation comes with a considerable burden of complications, leading to elevated patient morbidity, health care costs, and mortality [5-8]. The most recent consensus on device implantation and replacement by the European Heart Rhythm Association [9] has been significantly influenced by the invaluable contributions of various international registries. One of the most prominent among them is the “Replace” registry [10], which has played a pivotal role in advancing our understanding of this crucial aspect of cardiac care.

A comprehensive understanding of post-CIED implantation complications and their predictive factors is essential for patient management when considering CIED implantation. Therefore, a survey of patients undergoing CIED implantation in Tunisia is crucial to identify specific CIED complications due to the demographic and epidemiological specificities of the patients, as well as the conditions of the local health care system.

### Study Aims

This study aims to assess the incidence, characteristics, and predictors of complications arising in the initial 12 months following the implantation of CIEDs, describe the demographic and epidemiological profiles of a national cohort of consecutive patients with CIED, and provide insights into the level of compliance exhibited by Tunisian electrophysiologists with international guidelines pertaining to cardiac pacing and the prevention of sudden cardiac death.

## Methods

### Study Design and Patients’ Enrollment

NATURE-CIED (National Study of Cardiac Implantable Electronic Devices) is a nationwide, multicenter, prospective observational study with a 1-year follow-up period. The study’s steering committee extended invitations to all electrophysiologists, interventional cardiologists, and cardiovascular surgeons with experience in CIED implantation, regardless of whether they are practicing in the Tunisian public or private sectors, to participate in the study.

Patients’ enrollment occurred between January 18, 2021, and February 18, 2022, at all Tunisian public and private CIED implantation centers that agreed to participate in the study. Eligibility screening was conducted during hospitalization.

The study population consisted of consecutive patients undergoing primary CIED (conventional PMs, ICDs, and cardiac resynchronization: CRT-P–CRT-D) implantation, generator replacement, and upgrade procedure. To be enrolled, eligible patients have to provide written informed consent. All enrolled patients entered a 1-year follow-up period with 4 consecutive visits at 1, 3, 6, and 12 months after the CIED implantation. Exclusion criteria are nonconsenting patients and those operated exclusively for lead dysfunction.

### Data Sources

A steering board with 2 main investigators pre-established a specific case report form (CRF) for each type of CIED. Baseline data included patient demographics, ethnic specificity, medical history, cardiovascular history, cardiovascular risk factors, the CIED implantation indication, vital signs, laboratory measurements, electrocardiographic and echocardiographic findings, details of medical management, and results of eventual invasive and noninvasive investigations prior to CIED implantation (echocardiography, Holter monitoring, treadmill exercise stress test, and electrophysiological study).

The form also includes data regarding the technical aspects of the CIED implantation procedure and the occurrence of any periprocedural complications. Finally, the form specifies data on the clinical follow-up and device electrical testing parameters, as well as the eventual major incident events and CIED complications during the follow-up period. Investigators (electrophysiologists and interventional cardiologists) who agreed to participate in the study were asked to complete the pre-established form (CRF) for each type of implanted device at the enrollment visit and each scheduled visit.

Collected data were electronically recorded on a clinical suite platform (DACIMA software), which complies with international standards including US FDA 21 CRF part 11 (Food and Drug Administration 21 Code of Federal Regulations Part 11), US HIPAA (Health Insurance Portability and Accountability Act) and ICH (International Conference on Harmonisation), and Medical Dictionary for Regulatory Activities. The main investigators and the DACIMA consulting team periodically validated recorded data. The clinical suite platform generates alerts and requests in case of inconsistencies and missing data. Throughout the planned duration of the study, the steering board supervises the inclusion of patients, checks the data sources, and prepares the study’s statistical analysis plan.

### Timeline

**Figure 1:**
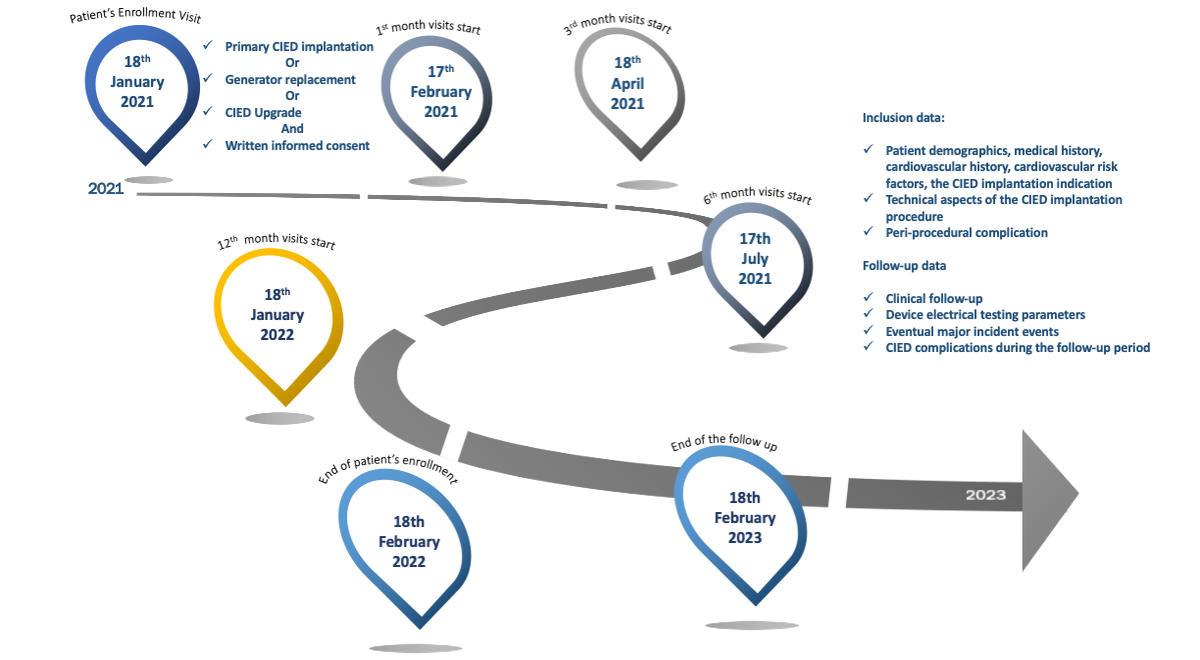
The Timeline of the Nature CIED study.

Patient enrollment and data collection began on January 18, 2021, and continued until February 18, 2022. Follow-up continued until all patients had 1-year data. The end of the study was therefore scheduled for February 18, 2023 (Figure 1).

If patients missed a scheduled follow-up visit, they or their relatives were contacted by phone to assess any adverse event and to reschedule a follow-up appointment.

### Outcomes

The main objective of this observational study is to assess the incidence, characteristics, and predictors of major and minor complications arising in the initial 12 months following the implantation of CIEDs.

The complications were characterized as major when life-threatening or requiring surgical reintervention for correction, and minor when suitable for treatment on an outpatient basis, involving device reprogramming, or requiring exclusive clinical observation [11]. Major complications include implantation failure, lead-related reinterventions, tamponade or myocardial perforation, pneumothorax requiring drainage, hematoma requiring evacuation, wound or pocket infection, systemic infections or endocarditis, Twiddler syndrome, and procedure-related deaths. Minor complications include pacing threshold increase, phrenic nerve stimulation, heart failure or left ventricular ejection fraction decrease, pericardial effusion, and conservatively treated hematoma and pneumothorax.

### Ethical Considerations

The study was performed according to the ethical principles for medical research involving human subjects specified in the Declaration of Helsinki and the ICH Good Clinical Practices. Ethics approval was obtained from the human research ethics committees at Abderrahmen Mami Hospital in Tunis (04/2021). Written informed consent was obtained from all the patients who participated in the study. The protocol of NATURE-CIED was approved by the Tunisian Society of Cardiology and Cardiovascular Surgery. The NATURE-CIED study was submitted to ClinicalTrials.gov (NCT05361759).

### Statistical Analysis

The DACIMA Clinical Suite platform enables the collection of web-based data and their extraction in SAS (SAS Institute) or SPSS (IBM Corp) format. The statistical analysis is exploratory, involving the calculation of 95% CI. The data will be described for the whole analyzed population. Continuous variables will be described by mean and SD or median and IQR. Categorical variables will be described by the size and frequency of every modality. Mean comparison will be performed by analysis of variance or by nonparametric tests if the hypothesis of normality is rejected.

The normality of continuous variables will be verified with the Shapiro-Wilk test. The statistical tests are bilateral with a 95% CI. A chi-square test will be performed for categorical variables. Yates correction or the Fisher exact test will be used if the conditions of validity for the chi-square test are not met.

A multivariate analysis will be performed with each complication as a dependent factor. The independent variables will be age, gender, ethnic specificity, BMI, CIED type, procedure type, procedure priority, implantation center (private and public), and CIED implanting operator whether he is an electrophysiologist or not (interventional cardiologist or cardiac surgeon).

Univariate logistic regression will be carried out with a 10% output threshold. The final model will be performed with the parameters selected by the backward stepwise method of Wald. The selected variables in the final model will be tested at the 5% threshold. The interaction between selected parameters is tested at the 10% threshold. The intermediate analyses will be carried out after formulating the statistical analysis plan.

### Expected Implications

NATURE-CIED is the first national and North African study that will (1) provide validated data on short- and medium-term complications and their predictors, which will allow us to individualize high-risk patients for optimal care, (2) identify the epidemiologic and demographic characteristics of Tunisian patients undergoing CIED implantation, and (3) clarify the contemporary CIED indications in Tunisia and their compliance with international guidelines.

## Results

In total, 27 investigators, comprising 14 from the public sector and 13 from the private sector, volunteered to take part in the study. It is worth noting that some of the investigators were involved in both the public and private sectors. A total of 1500 consecutive patients were enrolled for a 12-month follow-up period following CIED implantation. Sixty percent (n=900) of the patients were from the public sector, while 40% (n=600) were from the private sector. The patients’ mean age was 70.1 (SD 15.2) years, with a sex ratio of 1:15. A total of 1298 (86.3%) patients received a conventional PM and 75 (5%) patients received a biventricular pacemaker (CRT-P). ICDs were implanted in 127 (8.5%) patients. Of these patients, 45 (3%) underwent CRT-D implantation. The detailed analyses of this study are currently under investigation and will be published elsewhere in the near future.

## Discussion

### Summary

To the best of our knowledge, NATURE-CIED is the first National study of CIEDs in North Africa to provide the combination of large numbers of patients and devices and diverse implanting physicians. Twenty-seven cardiologists (electrophysiologists and interventional cardiologists) enrolled 1500 patients who underwent CIED implantation.

According to the second report from the Pan African Society of Cardiology working group on cardiac arrhythmias and pacing [12], Tunisia had the highest implant rates, with 164.3 PMs per million inhabitants and 2.16 PM implantation practitioners per million inhabitants in 2018, which makes it rank in the second position after Mauritius [12].

However, there is still a gap concerning patients’ demographics, CIED indications, and conformity to recent European Society of Cardiology guidelines [2] and CIED-related complications [12-14].

Both patient-related and procedure-related predictors of complications may identify patients with a particularly high risk of complications [15]. Previously reported incidences of complications after CIED implantation varied widely because of differences in the definitions of complications and length of follow-up [15-18]. Several previous studies in the 2000s reported that complication rates for PM were 4%-10% [13,17-19] and 3%-10% for ICDs and CTRDs [6,20]; however, an analysis of US Medicare states that data in 2014 showed much lower complication rates [21].

In 2010, the “Replace” registry, comprising data from a substantial cohort of 1744 patients who underwent device replacement procedures, yielded pivotal insights into predictive factors for complications [10]. This comprehensive analysis has, in turn, informed the development of practical and evidence-based procedural recommendations. By identifying the key determinants of complications, the registry has empowered health care professionals to more effectively anticipate and mitigate risks, ultimately leading to improved patient outcomes.

Therefore, the NATURE CIED study aims to offer detailed and comprehensive data on the predictors of CIED complications during the perioperative and 1-year follow-up period, which are derived from real-world data in Tunisia. The information generated by this study will prove valuable in the development of a national peri-implantation management protocol and the assessment of overall health care costs [11,12,14,22].

### Study Limitations

This study presents some limitations that must be considered in the interpretation of the results. This study is voluntary for both investigators and patients, and there is no routine audit to monitor the proportion of enrolled patients by participating electrophysiologists or cardiologists. This currently limits our ability to examine exact implant rates and to examine equity of access to PM therapy across the country. Some patients were lost to follow-up.

We identified all complications documented in patients during hospitalization and follow-up. We believe that this approach is the most accurate and comprehensive way to detect complications. In addition, this study was not designed to assess the effects of each investigator’s experience level and the volume of procedures performed individually. Therefore, the electrophysiologists’ or interventional cardiologists’ positions on their learning curve may influence the rate of complications mainly in teaching hospitals.

### Conclusions

NATURE-CIED study will fill an important gap in the management of patients undergoing CIED implantation both perioperatively and during follow-up and will identify patients at particularly high risk of complications for optimal management.

In Tunisia, this study will yield the largest contemporary longitudinal cohort of patients undergoing CIED implantation and will provide real-world data regarding heart rhythm diseases’ epidemiology and management with insights into the degree of adherence to international guidelines.

## Abbreviations

CIED: cardiac implantable electronic device
CRF: case report form
CRT-D: cardiac resynchronization therapy defibrillator
CRT-P: cardiac resynchronization therapy pacemaker
HIPAA: Health Insurance Portability and Accountability Act
ICD: implantable cardioverter defibrillator
ICH: International Conference on Harmonisation
NATURE-CIED: National Study of Cardiac Implantable Electronic Devices
PM: pacemaker
